# Automatic COVID-19 and Common-Acquired Pneumonia Diagnosis Using Chest CT Scans

**DOI:** 10.3390/bioengineering10050529

**Published:** 2023-04-26

**Authors:** Pedro Crosara Motta, Paulo César Cortez, Bruno R. S. Silva, Guang Yang, Victor Hugo C. de Albuquerque

**Affiliations:** 1 Department of Teleinformatics Engineering, Federal University of Ceará, Fortaleza 60455-970, Brazil; pedro.motta@lesc.ufc.br (P.C.M.); cortez@lesc.ufc.br (P.C.C.); bruno@lesc.ufc.br (B.R.S.S.); 2 National Heart and Lung Institute, Imperial College London, London SW7 2AZ, UK

**Keywords:** COVID-19, Computer-Aided Diagnostic, CNN, segmentation, classification, medical image, CT scan, external validation

## Abstract

Even with over 80% of the population being vaccinated against COVID-19, the disease continues to claim victims. Therefore, it is crucial to have a secure Computer-Aided Diagnostic system that can assist in identifying COVID-19 and determining the necessary level of care. This is especially important in the Intensive Care Unit to monitor disease progression or regression in the fight against this epidemic. To accomplish this, we merged public datasets from the literature to train lung and lesion segmentation models with five different distributions. We then trained eight CNN models for COVID-19 and Common-Acquired Pneumonia classification. If the examination was classified as COVID-19, we quantified the lesions and assessed the severity of the full CT scan. To validate the system, we used Resnetxt101 Unet++ and Mobilenet Unet for lung and lesion segmentation, respectively, achieving accuracy of 98.05%, F1-score of 98.70%, precision of 98.7%, recall of 98.7%, and specificity of 96.05%. This was accomplished in just 19.70 s per full CT scan, with external validation on the SPGC dataset. Finally, when classifying these detected lesions, we used Densenet201 and achieved accuracy of 90.47%, F1-score of 93.85%, precision of 88.42%, recall of 100.0%, and specificity of 65.07%. The results demonstrate that our pipeline can correctly detect and segment lesions due to COVID-19 and Common-Acquired Pneumonia in CT scans. It can differentiate these two classes from normal exams, indicating that our system is efficient and effective in identifying the disease and assessing the severity of the condition.

## 1. Introduction

Even with more than 80% of the population being wholly vaccinated against COVID-19, the disease still claims victims [1]. Moreover, the COVID-19 pandemic has caused several global economic, social, environmental, and healthcare impacts [2,3]. Computer-Aided Diagnostic (CAD) systems, which use machine learning methods to aid in the diagnostic process, can assist doctors in pinpointing specific areas of concern in medical images. These identified regions can then be used to detect illnesses and provide numerical data. Physicians can analyze these data to assess the progression or regression of the disease [4]. Thus, having a CAD system that can securely assist physicians in identifying SARS-CoV-2 and determining both the level of care required and whether the disease is progressing or digressing, particularly in the Intensive Care Unit (ICU), is crucial in the fight against this epidemic [5].

Computed Tomography (CT) scans of the chest can effectively diagnose individuals suspected of having SARS-CoV-2, as pneumonia is a frequently observed symptom of COVID-19 [6]. In CT analysis, the main characteristics present in patients with COVID-19 are ground-glass opacity (88.0%), bilateral involvement (87.5%), peripheral distribution (76.0%), and multilobar involvement (78.8%) [7]. Ground-glass opacity, seen on CT images as increased density in lung tissue, can be caused by various factors, including partial filling of the alveoli, increased blood flow, or a combination of both. While it is a common finding in CT scans of individuals diagnosed with COVID-19, it is not exclusive to the virus. Other conditions, such as influenza, cytomegalovirus, Common-Acquired Pneumonia (CAP), and pulmonary edema, can also cause it. Therefore, solely relying on detecting and segmenting ground-glass opacity is not a sufficient method for diagnosing COVID-19 [8].

Therefore, this paper aims to provide a ready-to-use pipeline that segments ground-glass opacity and consolidation lesions on full CT scans, classifies exams with COVID-19 or Common-Acquired Pneumonia, and quantifies the severity of lesions on full COVID-19 CT scans. The following research questions are then stated:

**RQ1.**  Is there a difference in results of state-of-the-art models for lung and lesion segmentation?
  This question aims to statistically analyze the results obtained with our architectures to validate their relevance.
**RQ2.**  Can lesion segmentation architectures detect COVID-19 and CAP in an external validation dataset?
  Many machine learning architectures are biased to the dataset distribution they are trained on and do not achieve satisfactory results when externally tested on a dataset with a different distribution.
**RQ3.**  Is a single slice from a full CT scan sufficient for differentiating COVID-19 and CAP?
  Our pipeline aims to quickly and efficiently provide a diagnosis suggestion as to whether a CT scan is from a patient with COVID-19, CAP, or neither. Thus, we explore the possibility of reducing the classification processing time from a 3D CT scan to a 2D image slice.
**RQ4.**  Is it possible to determine COVID-19 severity only by quantifying segmented lesions?
  As COVID-19 has a range of severity levels and each severity level might need a different treatment, healthcare professionals must have quantitative data on lung involvement.

Thus, the main contributions of this work are listed below:

- A complete pipeline for segmenting lungs and lesions, detecting COVID-19 and CAP, and calculating COVID-19 severity;
- An extensive segmentation architecture statistical analysis on a combination of datasets with healthy patients, COVID-19 patients, and patients affected by other diseases for lung and lesion detection;
- A cross-dataset approach, aiming at better model generalization using an external validation dataset.

This work is organized as follows: Section 2 provides a revision of related works in the literature. In Section 3, we describe our applied methodology. The results and discussion are presented in Section 4. Finally, the conclusions of this work are detailed in Section 5.

## 2. Related Works

Because of the rapid manifestations of COVID-19 and the significant number of disease cases, many Artificial Intelligence (AI) studies have been conducted to aid medical diagnosis with medical data in the areas of disease classification, and lung and lesion segmentation. We selected novel works published in journals of relevant impact that presented similarity to our work in materials, such as datasets and architectures, or scope and methodology.

Natural Language Processing models can efficiently extract information from clinical reports, providing a comprehensive view of a patient’s symptoms and medical history. NLP models can be helpful in scenarios where radiographic images are unavailable or difficult to obtain. Moreover, NLP models can be trained on relatively small datasets, which may be beneficial when data availability is limited. On the other hand, image analysis with CNNs can provide more direct and accurate information about the presence of COVID-19 in radiographic images. CNNs have shown great promise in accurately detecting COVID-19 in chest X-rays and CT scans. However, CNNs require large datasets to be trained effectively, and interpreting the results may not always be straightforward.

Some authors applied NLP methods to extract text information from medical reports to identify evidence of COVID-19 [9] by analyzing symptoms such as fever, cough, headache, fatigue, dyspnea, and others in 359,938 patients with laboratory tests positive for SARS-CoV-2. Others performed text classification based on radiology or CT scan reports [10,11] to classify COVID-19 and non-COVID-19 patients.

The use of machine learning in COVID-19 detection with X-ray imaging has been explored in scientific research. Many papers have proposed using algorithms, such as traditional machine learning, Convolutional Neural Networks (CNNs), and transfer learning, to analyze X-rays to detect the disease. These studies have shown promising results, demonstrating the potential of machine learning in aiding clinicians in their screening process and improving the speed and accuracy of COVID-19 diagnosis.

Some works have proposed different methods to detect COVID-19 using X-ray images. For example, while Ohata et al. [12] and Basha et al. [13] used machine learning methods for feature extraction and classification, Hu et al. [14] employed transfer learning and pre-trained models. Despite the promising results obtained by these studies, some limitations could still be addressed. For example, the studies employed relatively small datasets, which may limit their generalizability. Nonetheless, all three papers are limited by the resolution of the X-ray images, which can affect detection accuracy.

Machine learning has also been used to detect COVID-19 in CT images. This approach is considered more sensitive than traditional methods such as X-rays and PCR, as CT scans provide high-resolution images more suited to analysis using machine learning algorithms [6,15]. Furthermore, using machine learning in CT images also aids human interpretation, which can be prone to errors and subjectivity. Hence, combining machine learning to assist in COVID-19 diagnosis with CT images is a promising development in the fight against the pandemic.

Overall, previous papers demonstrated the potential of deep learning models for detecting and classifying COVID-19 using CT scans. They used different architectures, pre-processing techniques, and datasets to achieve their results, showing promising results in distinguishing COVID-19 from healthy or CAP patients. Some developed new architectures, such as AH-Net, ReCOV-101, and COVNet [16,17,18], while others used transfer learning techniques [19,20]. However, these studies also had limitations, as they only classified CT scans, or even single slices, in classes such as normal and COVID-19; normal, COVID-19, and CAP; COVID-19 and non-COVID-19; and normal and COVID-19 severity. They lacked the usage of an external validation dataset. In addition, some used explainability algorithms to interpret the classifications made by the models [16,18], which are still unreliable according to doctors [21,22], and none returned quantitative values.

Several papers proposed deep learning techniques to segment and classify COVID-19 pneumonia lesions in CT scans. Zhang et al. [23] adapted 3D ResNet-18 to segment lesions. Amyar et al. [24] developed a Multi-Task Learning (MTL) architecture based on COVID-19 classification, lesion segmentation, and image reconstruction. Qiblawey et al. [25] used encoder–decoder CNNs, UNet, and FPN to segment the lungs and COVID-19 lesions, achieving high COVID-19 detection performance. Wang et al. [26] proposed a noise-robust Dice loss function and a self-ensembling framework for COVID-19 lesion segmentation. Finally, Zhou et al. [27] used a CT scan simulator for COVID-19 and a deep learning algorithm to segment and quantify the infection regions.

These works mainly segmented lesions and classified exams as COVID-19 or normal, providing more quantitative results than classification models and explainability algorithms. However, if CAP exams were provided to their models, these exams were wrongfully classified as COVID-19 [25,26,27]. Amyar et al. conducted lesion segmentation and classification but did not validate their methods on an external dataset [24].

Zhang et al. [23] segmented the whole CT scan for both lungs and lesions and then forwarded the full CT scan to a 3D ResNet, a 3D network that takes longer to train and evaluate than our 2D approach, which selects one slice from the full CT scan to classify. Moreover, they did not make their full dataset publicly available, which makes validation and comparison difficult.

Diagnosis involves identifying a disease or condition based on signs, symptoms, and diagnostic tests, while prognosis involves predicting the likely course of a disease or condition and its possible outcomes. In various medical applications, deep learning models have been used in diagnosis and prognosis tasks. Some works focused on developing deep learning models for accurate disease diagnosis [12,13,14,16,18,24,26]. In contrast, other works focused on predicting the prognosis of a disease [17,23,25,27], such as estimating the likelihood of survival or disease progression. Finally, some papers combined both diagnosis and prognosis tasks. Our work aims to do both, i.e., diagnosing the disease as COVID-19 or CAP, and if the disease is classified as COVID-19, giving the prognosis of the severity of the disease. While deep learning models have shown promise in both diagnosis and prognosis tasks, it is essential to recognize the limitations of these models and use them in conjunction with other clinical information and expertise [28,29].

A brief comparison between CT scan-related papers and this work can be found in Table 1. We highlight that we made a complete pipeline for segmenting lesions, detecting disease, and then classifying the full CT scan as COVID-19 or CAP in an external dataset using only public data.

## 3. Materials and Methods

**Figure 1:**
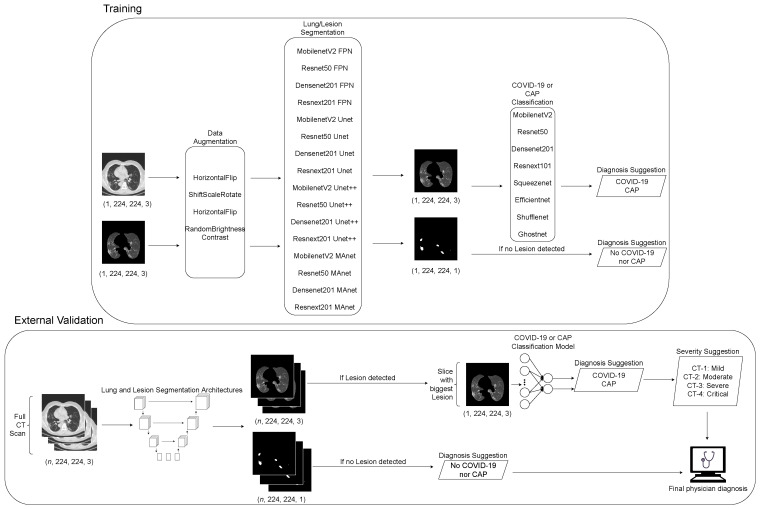
Fluxogram of proposed pipeline employed in this work.

This section provides a description of the workflow of this work. We first merged public datasets from the literature to train lung and lesion segmentation models with different distributions. Then, we trained classification CNN models on a subset of the COVIDxCT dataset, containing only COVID-19 and CAP classes. Finally, if the exam was classified as COVID-19, we quantified the lesions and evaluated the severity of the exam using MosMedData. We applied our entire pipeline to the SPGC dataset, dividing it into normal and lesion exams and secondly into COVID-19 and Common-Acquired Pneumonia lesions. The workflow flowchart can be seen in Figure 1.

As CT scans can have *n* different numbers of slices, we applied our segmentation models to all slices. First, the exam was classified as normal if no lesion was detected on the slices. Next, the exam was classified as “with lesions” if lesions were detected. Then, the slice with the biggest lesion area was used to classify the whole exam as COVID-19 or CAP.

### 3.1. Datasets

We employed a combination of three public datasets for the lung segmentation task, resulting in a total of 3677 images from chest CT scans and their corresponding lung masks [30,31,32]. For the lesion segmentation task, we utilized a combination of four public datasets, yielding 6493 images from chest CT scans and their lesion masks [30,31,32,33]. Both tasks employed 10-fold cross-validation, with a 80%–20% split for training and testing, respectively, and 10% of the training data were allocated for validation. To ensure consistency, we transformed all images from the DICOM or NIFTI format into PNG in the Hounsfield unit range of 0–255 using a window of −500 and a width of 750.

For the classification task, we utilized the COVIDxCT dataset, which included 294,552 images from COVID-19-positive cases and 62,966 images from Common-Acquired Pneumonia cases for training; in total, 8147 and 8008, respectively, were used for validation; and 7965 and 7894, respectively, were used for testing. As the COVIDxCT dataset already provides a set train/validation/test split, we did not use k-fold cross-validation to conduct a comparison with benchmarks [34].

MosMedData provides 50 COVID-19-positive CT scans with lesion segmentation golden standard. We randomly selected 50 COVID-19-negative exams to add 100 MosMedData exams to our training set. Then, we used the remaining 1010 exams to validate our lesion quantification and disease severity step. MosMedData has an average of 42 slices per exam. COVID-19 scans are divided into four classes: CT-1 to CT-4, with increasing severity, and CT-0, the COVID-19-negative class. Samples are distributed as follows: CT-0—254; CT-1—684; CT-2—125; CT-3—45; CT-4—2 [33].

Finally, we employed the SPGC dataset for external validation to answer **RQ3**; the dataset includes 307 full CT scans, where 76 are of normal patients, 60 are of Common-Acquired Pneumonia patients, and 171 are of COVID-19 patients. Each exam has an average of 150 slices [35]. Table 2 summarizes all datasets used in this work and the task they were used for.

### 3.2. Data Augmentation

To expand the generalization capabilities of our models and produce more images with lesions, on our training sets, we used data augmentation methods such as randomly flipping the image horizontally; randomly translating, scaling, and rotating the image; randomly shifting values for each channel of the input RGB image; and randomly changing the brightness and contrast of the image [36]. Table 3 shows a summary of the techniques and parameters.

### 3.3. Grid Search

As lesion segmentation is a more complex task than lung segmentation, we used grid search for 200 runs to optimize our hyperparameters and obtain better results with each architecture [37]. Table 4 shows a summary of the optimized hyperparameters and their parameters.

### 3.4. Segmentation Models

We utilized well-known, state-of-the-art, and novel encoders and decoders to analyze various structures for lung and lesion segmentation [38]. We tested sixteen combinations of encoders and decoders, combining methods with different sizes and techniques as displayed in Table 5. The encoders utilized in this evaluation were MobilenetV2, Resnet50, Densenet201, and Resnext101 [39,40,41,42]. The decoders used were Unet, FPN, Unet++, and MAnet [43,44,45,46].

The chosen loss function for lung segmentation was Lovasz, and the learning rate was set to 0.001 with Adam optimization. The batch size was 64, and the maximum number of epochs was 50. For lesion segmentation, we optimized each hyperparameter for the F1-score metric with grid search, and the values are presented in Table 5.

Tversky loss is a loss function that is commonly used in machine learning for binary classification problems where the classes may not be balanced, such as our lesion segmentation task. It is a generalization of the Dice loss. It is the only loss from the selected ones with the possibility of defining a β-value choice to tune the desired trade-off between false positives and false negatives [47].

### 3.5. Lesion Quantification

MosMedData does not provide a quantified computed approach for severity analysis; expert physicians qualitatively evaluate COVID-19 severity. First, we calculated the area of the left and right lungs and lesions to approximate the severity analysis. Then, we divided the area of the lesions by the area of the lung in which they were to obtain the percentage of parenchymal involvement for each lung. Finally, to answer **RQ2**, we used thresholds of 0<x≤25, 25<x≤50, 50<x≤75, and x>75, where *x* is the percentage of parenchymal involvement for a lung.

### 3.6. Classification Models

We tested eight state-of-the-art models to answer **RQ4**, including MobilenetV2, Resnet50, Densenet201, Resnext101, Squeezenet, Efficientnet, Shufflenet, and Ghostnet, all pre-trained on ImageNet. The loss function utilized was cross-entropy; the learning rate was set at 0.0001; and Adam optimization was used. The batch size was 64; the maximum number of training epochs was 20; and patience was 5 epochs.

### 3.7. Evaluation Metrics

Segmentation models were evaluated using accuracy, F1-score (DSC score), Hausdorff Distance (HD), and training and testing time. Classification models were evaluated using accuracy, F1-score, precision, recall, specificity, and confusion matrix.

For the segmentation tasks, true positive refers to correctly segmented lesion pixels; true negative, to correctly segmented background pixels; false positive, to background pixels wrongfully classified as lesion pixels; and false negative, to lesion pixels wrongfully classified as background pixels.

For the classification tasks, true positive refers to correctly classified exams with lesions; true negative, to correctly classified exams without lesions; false positive, to exams without lesions wrongfully classified as “with lesions”; and false negative, to exams with lesions wrongfully classified as “without lesions”.

(1) $$\mathrm{Acc}\;=\;\frac{\mathrm{TP}+\mathrm{TN}}{\mathrm{TP}+\mathrm{TN}+\mathrm{FP}+\mathrm{FN}}$$

Higher accuracy mainly indicates better performance. However, accuracy is not always the best metric to evaluate a model, mainly because we are dealing with imbalanced data, and misclassifications have different consequences. For example, it is worse to classify a COVID-19 exam as a non-COVID-19 exam than the other way around.

(2) $$F1\;=\;\mathrm{DSC}\;=\;\frac{2\mathrm{TP}}{2\mathrm{TP}\;+\;\mathrm{FP}\;+\;\mathrm{FN}}$$

F1-score, on the other hand, is a metric that considers both precision, where high precision indicates that the model accurately identifies positive cases,

(3) $$P\;=\;\frac{\mathrm{TP}}{\mathrm{TP}+\mathrm{FP}}$$

and recall, where high recall indicates that the model accurately identifies most positive cases, even if it also misclassifies some negative cases as positive,

(4) $$R\;=\;\frac{\mathrm{TP}}{\mathrm{TP}+\mathrm{FN}}$$

F1-score is the harmonic mean of precision and recall and provides a balance between the two metrics. In cases where the data are imbalanced, F1-score can provide a more informative evaluation of the model’s performance, because it penalizes models that only predict the majority class. Therefore, as our goal is to identify COVID-19-positive cases with high precision, F1-score may be a more appropriate metric than accuracy.

Specificity refers to the ability of a model to correctly identify the negative cases, i.e., those that do not have COVID-19. High specificity indicates that the model can accurately identify people who do not have the virus, which is essential to avoid false positives.

(5) $$S\;=\;\frac{\mathrm{TN}}{\mathrm{TN}+\mathrm{FP}}$$

It is important to note that a model with low specificity but high recall identifies many true-positive cases but also has many false positives. Finally, segmentation models were also evaluated using Hausdorff Distance (HD):

(6) $$d\left(X,Y\right)\;=\;\sup\left\{\underset{x\in X}{\sup}\underset{y\in Y}{\inf}d\left(x,y\right),\underset{y\in Y}{\sup}\underset{x\in X}{\inf}d\left(x,y\right)\right\}.$$

Hausdorff Distance is a metric on the space of compact, non-empty sets. The Hausdorff metric between two sets, X and Y, is defined as the maximum of two values: the Hausdorff Distance from X to Y and the Hausdorff Distance from Y to X. The Hausdorff metric is commonly used in computer vision, image processing, and pattern recognition. It compares the similarity of shapes, images, or other data types. In this work, X and Y are the segmented images returned by our architectures and the ground-truth images, respectively.

### 3.8. Statistical Tests

We used boxplots for visualizing and comparing the distributions of numerical data. They provide a quick summary of the data’s central tendency, spread, and skewness and can be particularly useful for identifying outliers and skewness in the data.

To better understand the significance of our results, meaningfully analyze the best models, and answer **RQ1**, we used the following steps for statistical analysis:

1. All columns were checked with the Shapiro–Wilk test for normality.
2. If all columns were normal, we used Bartlett’s test for homogeneity; otherwise, we used Levene’s test.
3. If all populations were normal and homoscedastic, we used repeated measures ANOVA with Tukey’s HSD as post hoc test.
4. If at least one population was not normal or the populations were heteroscedastic, we used Friedman’s test with the Nemenyi post hoc test.

We used the Shapiro–Wilk test to test the normality assumption [48]. Then, we applied the Bartlett’s or Levene’s test, depending on the Shapiro–Wilk’s results.

Bartlett’s test is a homogeneity test of variances to determine if the variances of the metrics of the architectures are equal. It tests the null hypothesis that the variances of all groups are similar [49].

Levene’s test assesses the assumption of equal variances before conducting a test to compare the means of the metrics of the architectures. It provides a way to determine if the variances of the groups (each group is a 10-fold result for a metric) are equal, which is an essential assumption for the ANOVA test [50].

We conducted the repeated measures ANOVA test to determine if there was a significant difference in the means of the metrics of the architectures. The repeated measures ANOVA test calculates a statistic and provides a p-value, which can be used to determine if the differences among the group means are significant [51].

We performed Friedman’s non-parametric test to determine if there was a significant difference among the metrics of the architectures. It tests the null hypothesis that the population medians of all groups are equal. The Nemenyi post hoc test is a multiple comparison test that we used to identify which groups were significantly different from each other after a significant result of Friedman’s test [52,53].

Tukey’s HSD test is a multiple comparison test used to compare all possible pairs of means in the set of metrics of the architectures. We used Tukey’s HSD test to identify which specific pairs of metrics were significantly different from each other, considering the multiple comparisons. We utilized Bartlett’s test to assess the assumption of equal variances before conducting Tukey’s HSD test. If Bartlett’s test shows that the variances are equal, then Tukey’s HSD test can be used to compare the means of the metrics of the architectures [54].

### 3.9. Development Environment

For the development of this work, we utilized several cutting-edge tools and technologies to ensure the best possible outcome. We employed PyTorch, Pytorch Lightning, Segmentation Models Pytorch (SMP), Autorank, Pytorch GradCAM [55], and WandB. Our hardware setup included an NVIDIA GeForce RTX 3060 12 GB graphics card and a 12th Gen Intel Core i7-12700KF x 20 processor, along with 64 GB of memory.

## 4. Results and Discussion

This section presents the results concerning the methodology employed for lung and lesion segmentation, and COVID-19 and CAP classification in CT exams. We compared state-of-the-art models using accuracy, precision, recall, F1-score, specificity, Hausdorff Distance, and processing time.

### 4.1. Lung Segmentation

The first task was to segment the lungs from the background on raw CT slices to remove unnecessary artifacts for COVID-19 and CAP detection. We summarize the results of this step in Table 6.

In general, all architectures presented excellent results regarding accuracy, F1-score (DSC), and Hausdorff Distance. Resnext101 Unet++ outperformed the other architectures in all metrics, achieving 99.71 ± 0.05%, 98.64 ± 0.19%, and 3.9 ± 0.16 in accuracy, F1-score (DSC), and Hausdorff Distance, respectively. However, all architectures presented a similar performance in the three metrics. In the following sections, we analyze the significance of our results with statistical tests, aiming to confirm their relevance.

**Figure 2:**
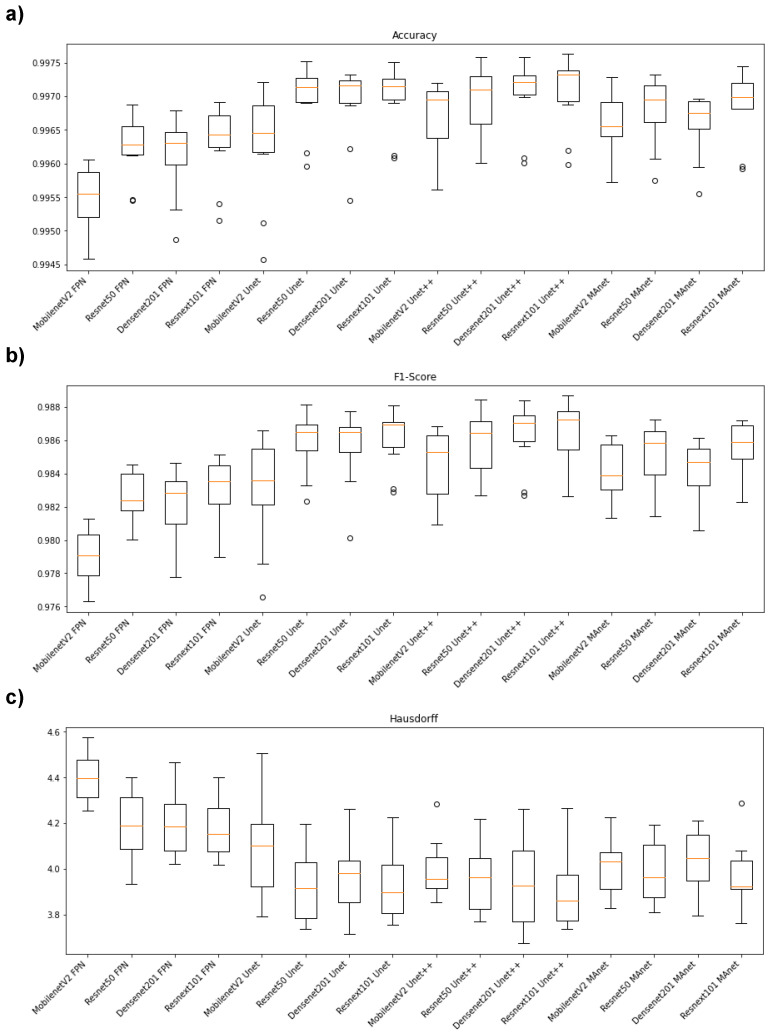
Boxplots of segmentation metrics applied in this work. (**a**) Accuracy, (**b**) F1-score (DSC), and (**c**) Hausdorff Distance.

Figure 2 illustrates the segmentation metric boxplots applied for lung segmentation: accuracy, F1-score (DSC), and Hausdorff Distance.

Concerning the accuracy metric, we can see, by considering the *y*-axis, that all algorithms performed similarly, as accuracy varied from 0.9945 to 0.9975. First, however, we remark on some important aspects when comparing our segmentation architectures. For instance, Resnet50 Unet, Densenet201 Unet, Resnext101 Unet, Densenet201 Unet++, and Resnext101 Unet++ presented the best accuracy medians (Figure 2a), lying higher than other algorithm boxes. Moreover, the interquartile ranges of these algorithms were smaller than those of the others, indicating that the accuracy values were less dispersed with a left-skewed distribution. On the other hand, MobilenetV2 FPN presented the lowest accuracy with more dispersed data and a soft left-skewed distribution. The remaining algorithms presented competitive accuracy results but with dispersed and skewed values. In addition, only MobilenetV2 FPN, MobilenetV2 Unet++, Resnet50 Unet++, and MobilenetV2 MAnet had no outliers.

In general, the F1-score behavior was similar. For example, Resnet50 Unet, Densenet201 Unet, Resnext101 Unet, Densenet201 Unet++, and Resnext101 Unet++ again presented the best median values (Figure 2b), with a left-skewed distribution. However, Resnet101 Unet++ had a more dispersed data distribution.

The architectures had more dispersed data for the Hausdorff metric (Figure 2c). For example, Resnext101 Unet++ had the lowest median, with a right-skewed distribution, and MobilenetV2 FPN presented the highest Hausdorff median.

**Figure 3:**
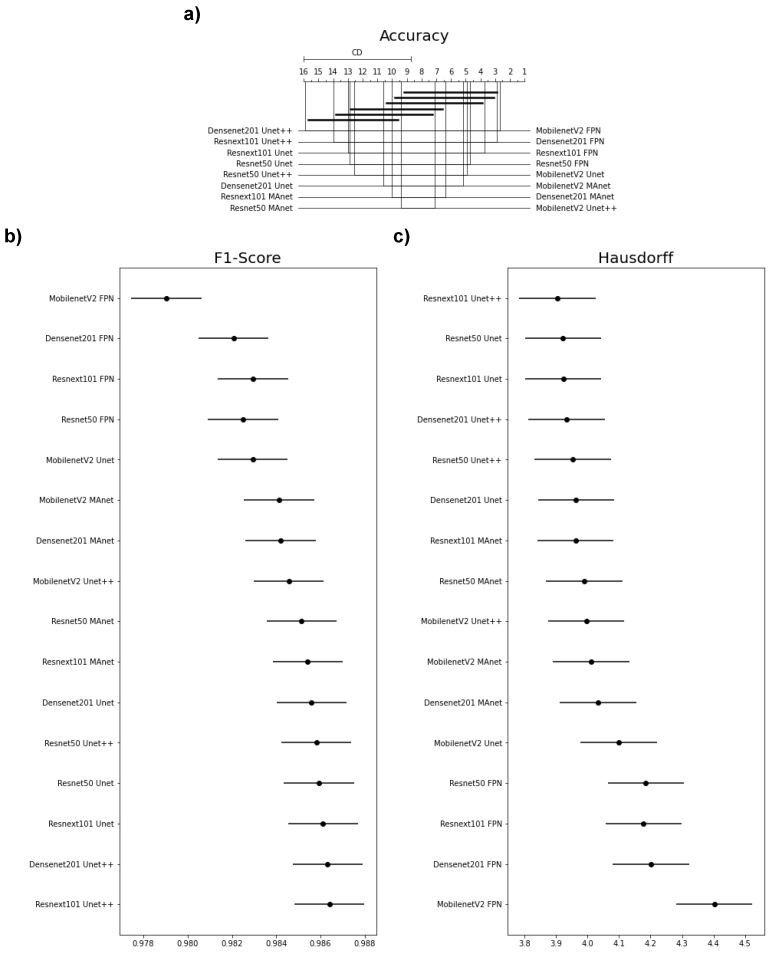
Statistical test results of our metrics for the lung segmentation task. (**a**) Accuracy, (**b**) F1-score (DSC), and (**c**) Hausdorff Distance.

Because one accuracy population was not normal (Densenet201 Unet), we applied Friedman’s test with the Nemenyi post hoc test to analyze whether the distributions of the accuracy results differed. We present the test results in Figure 3a. Differences are significant if the distance between the mean ranks is greater than the Critical Distance (CD).

We failed to reject the null hypothesis that the population was normal for all F1-score populations. Therefore, we assumed that all F1-score populations were normal. We applied Bartlett’s test for homogeneity and failed to reject the null hypothesis that the data were homoscedastic. Thus, we assumed that our data were homoscedastic. Because we had more than two populations and all populations were normal and homoscedastic, we used repeated measures ANOVA as an omnibus test to determine any significant differences among the mean values of the populations. As the results of the ANOVA test were significant, we used Tukey’s HSD post hoc test to infer which differences were significant. Populations were significantly different if their confidence intervals were not overlapping; see Figure 3.

None of the architectures significantly differed in accuracy, as they had a mean rank distance smaller than the Critical Distance for at least one other evaluated architecture (Figure 3a). Nonetheless, the architecture that had the most different accuracy from the others was MobilenetV2 FPN.

**Figure 4:**
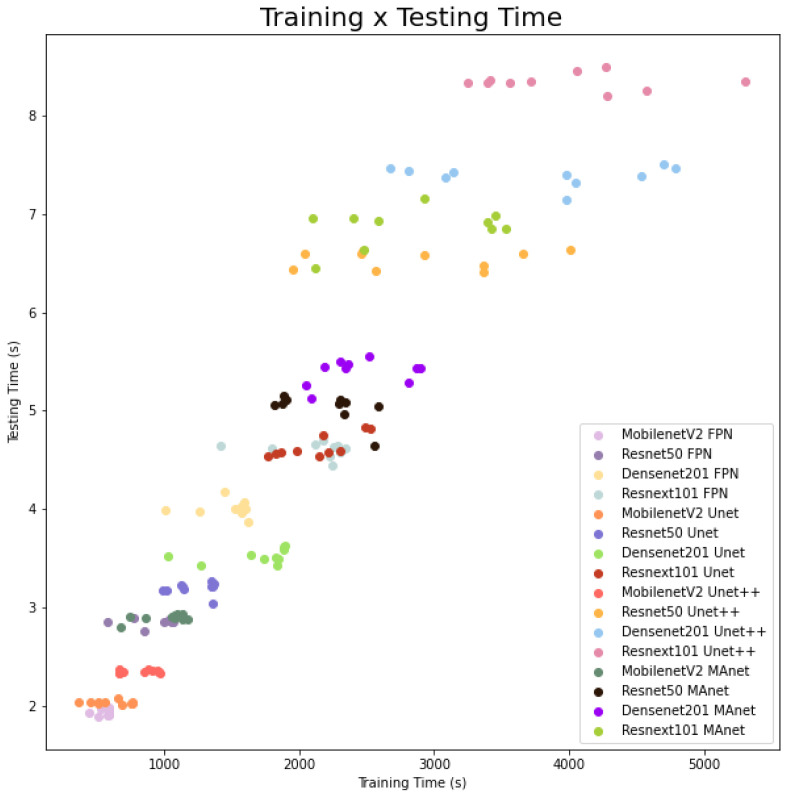
Training and testing time for lung segmentation.

Most confidence maps overlapped (Figure 3b), except for MobilenetV2 FPN, the fastest architecture in training and testing (Figure 4). When selecting an architecture, we can choose MobilenetV2 FPN for a fast architecture with a slight loss in F1-score. On the other hand, let us suppose that we decide on an architecture with higher F1-score. In that case, we can choose any other architecture, because F1-score differences are insignificant. Thus, the best choice would be Resnet50 Unet++, the second fastest architecture, which, as shown by the test, did not significantly differ in F1-score from other slower architectures.

The Hausdorff Distance results were generally similar (Figure 3c). Again, MobilenetV2 FPN had the most significant difference, while other architectures had no significant difference in Hausdorff Distance.

The fastest model for training and testing was MobilenetV2 FPN, and the slowest one was Resnext101 Unet++. However, even if the shortest training time (513.5 s) was more than ten times faster than the longest training time (5304.3 s), the fastest testing time was 1.9 s, and the slowest testing time was 8.5 for evaluating 3677 images, or averages of $0.51\times 10^{-3}$ and $2.3\times 10^{-3}$ s per image, respectively. As complexity increased, other models followed linear training and testing time growth. We present this behavior in Figure 4.

### 4.2. Lesion Segmentation

The second task was to segment lesions inside the lungs from the previously segmented CT slices for COVID-19 and CAP detection. We summarize the results of this step in Table 7.

All architectures presented excellent results regarding accuracy, F1-score (DSC), and Hausdorff Distance. Densenet201 Unet, Resnet50 Unet++, and Resnext101 Unet++ outperformed the other architectures in accuracy, achieving 99.87±0.01%. Densenet201 Unet++ obtained the highest F1-score (DSC) among all architectures, achieving 85.16±1.13%. However, all architectures presented a similar performance in the three metrics. In the following sections, we analyze the significance of our results with statistical tests, aiming to confirm their relevance. However, MobilenetV2 FPN, the fastest architecture, obtained the smallest HD of 2.86±0.12.

The accuracy results were high because most of the ground-truth image was composed of black pixels, with only a small percentage of the image being white lesion pixels. When we calculated the accuracy of our models, these black pixels increased all accuracy results, reducing the metric credibility.

**Figure 5:**
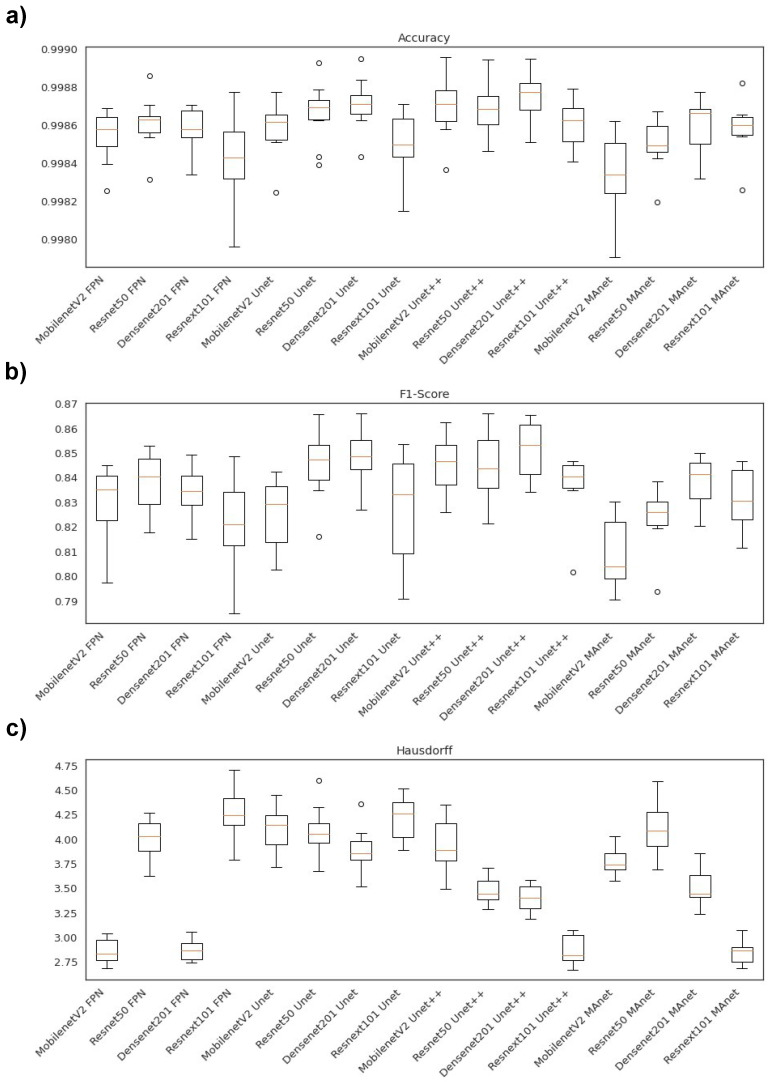
Boxplots of segmentation metrics applied in this work for lesion segmentation. (**a**) Accuracy, (**b**) F1-score(DSC), and (**c**) Hausdorff Distance.

Figure 5 illustrates the segmentation metric boxplots applied for lesion segmentation: accuracy, F1-score (DSC), and Hausdorff Distance.

Concerning the accuracy metric, we can see, by considering the *y*-axis, that all algorithms performed very similarly, as accuracy varied from 0.9980 to 0.9990. First, however, we remark on some important aspects when comparing our segmentation architectures in terms of this metric. For instance, Resnet50 Unet, Densenet201 Unet, Densenet201 Unet++, and Resnext101 Unet++ presented higher accuracy medians (Figure 5a). Moreover, the interquartile ranges of these algorithms were smaller than those of the others, indicating that the accuracy values were less dispersed with a left-skewed distribution.

On the other hand, MobilenetV2 MAnet presented the lowest accuracy, with more dispersed data and a soft left-skewed distribution. The remaining algorithms presented competitive accuracy results but with more dispersed and skewed values. In addition, only Densenet201 FPN, Resnext101 Unet, Resnet50Unet++, Densenet 201 Unet++, Resnext101 Unet++, and Densenet201 MAnet had no discrepant values.

Concerning F1-score, the Unet decoders ( Resnet 50 Unet, Densenet201 Unet, MobilenetV2 Unet++, Resnet50 Unet++, and Densenet201 Unet++) presented higher median values with lower dispersion (Figure 5b). On the other hand, Resnext101 Unet had a more dispersed data distribution. Moreover, only the Resnet50 Unet, Resnext101 Unet++, and Resnet50 MAnet architectures presented discrepant values.

In general, the architectures had less dispersed data for the Hausdorff metric (Figure 5c). For example, Resnext101 Unet++ had the lowest median, with a right-skewed distribution, and MobilenetV2 FPN, Densenet201 FPN, Resnext101 Unet++, and Resnext101 MAnet presented the lowest Hausdorff median.

**Figure 6:**
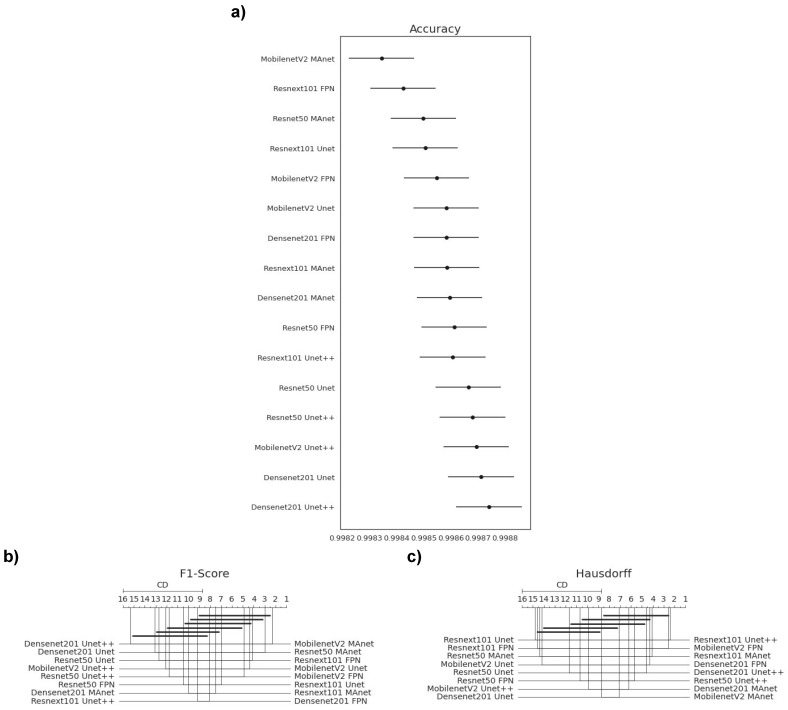
Statistical test results of our metrics for the lesion segmentation task. (**a**) Accuracy, (**b**) F1-score, and (**c**) Hausdorff Distance.

We failed to reject the null hypothesis that the population was normal for all accuracy populations. Therefore, we assumed that all accuracy populations were normal. We applied Bartlett’s test for homogeneity and failed to reject the null hypothesis that the data were homoscedastic. Thus, we assumed that our data were homoscedastic. Because we had more than two populations and all populations were normal and homoscedastic, we used repeated measures ANOVA as an omnibus test to determine any significant differences among the mean values of the populations. As the results from the ANOVA test were significant, we used Tukey’s HSD post hoc test to infer which differences were significant. Populations were significantly different if their confidence intervals were not overlapping; see Figure 6a.

Because one F1-score and one HD population were not normal (Resnext101 Unet++), we applied Friedman’s test with the Nemenyi post hoc test to analyze if there was a difference among the distributions of the accuracy results. We present the test results in Figure 6b,c. Differences were significant if the distance between the mean ranks was greater than the Critical Distance (CD).

Most confidence maps overlapped (Figure 6a), except for MobilenetV2 MAnet, which mainly overlapped with Resnext101 FPN and Resnet50 MAnet. Resnext101 FPN and Resnet50 MAnet had similar results in all metrics and similar training and testing times. However, MobilenetV2 MAnet was faster for training and testing, with a small decrease in accuracy (Figure 4). Thus, when selecting an architecture, we can choose MobilenetV2 MAnet for a fast architecture with a slight loss in accuracy. On the other hand, let us suppose that we decide on an architecture with higher accuracy. In that case, we can choose any other architecture, because F1-score differences are insignificant. Thus, the best choice would be Resnet50 Unet++ again, the second fastest architecture, which, as shown by the test, did not significantly differ in F1-score from other slower architectures.

None of the architectures significantly differed from the others in F1-score, as they had a mean rank distance smaller than the Critical Distance for at least one other evaluated architecture (Figure 6b). Nonetheless, the architecture that had the most different F1-score from the others was MobilenetV2 MAnet.

In general, the Hausdorff Distance results were similar (Figure 6c). MobilenetV2 FPN, Resnext101 Unet++, and Resnext101 MAnet had the most significant difference, while other architectures had no significant difference in Hausdorff Distance.

**Figure 7:**
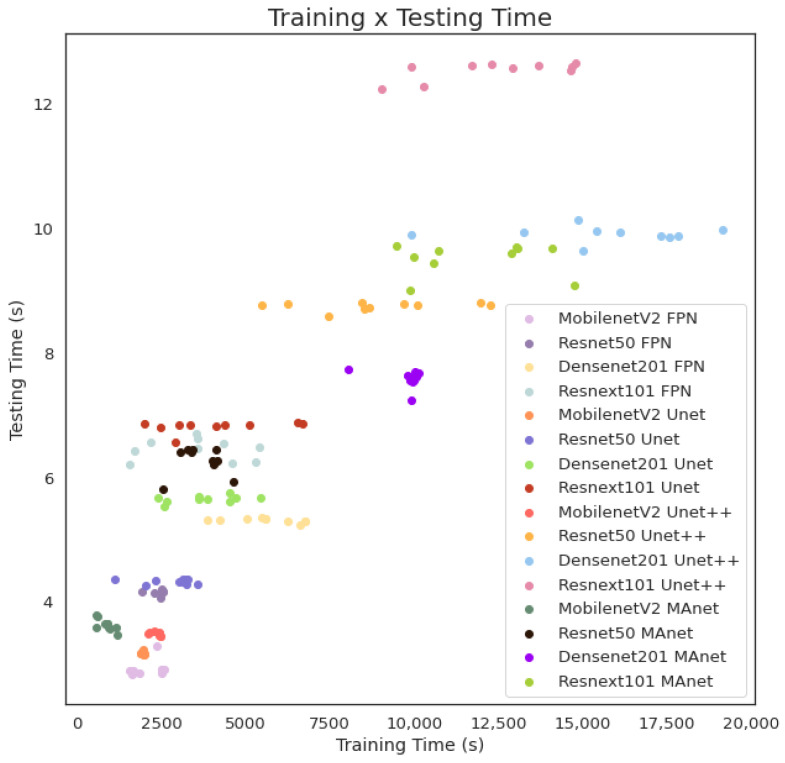
Training and testing time for lesion segmentation.

The fastest model for training was MobilenetV2 MAnet, and for testing, it was MobilenetV2 FPN. However, MobilenetV2 MAnet converged faster, needing only 25 epochs. The slowest ones for training were Densenet201 Unet++ and Resnext101 Unet++, and the slowest for testing was Resnext101 Unet++. However, even if the fastest training time (573.0 s) was more than thirty times faster than the slowest training time (19,164.5 s), the fastest testing time was 2.8 s, and the slowest testing time was 12.6 s for evaluating 6493 images, or averages of $0.43\times 10^{-3}$ and $1.9\times 10^{-3}$ s per image, respectively. As complexity increased, other models followed linear training and testing time growth. We present this behavior in Figure 7.

Finally, we answer **RQ1**, as we have shown that all architectures mainly had similar results in lung and lesion segmentation, without statistical differences in metrics, and achieved competitive results. The main differences were in training and testing time. MobilenetV2 FPN was the fastest for lung segmentation training and testing; MobilenetV2 FPN, for lesion segmentation training; and MobilenetV2 MAnet, for lesion segmentation testing.

### 4.3. Lesion Detection

We first applied our architectures to the other 1010 full CT scans of MosMedData to validate our pipeline in a 3D scenario to detect and segment all lesions in an exam and then classify the exam as “with lesion”, if any lesion was found, or “without lesion”, otherwise. The results are summarized in Table 8.

All architectures had similar and competitive results on MosMedData. Mobilenet Unet had the highest accuracy, F1-score, and recall, with 94.36%, 96.5%, and 97.39%, respectively. However, it only achieved the specificity of 82.35%. Densenet201 MAnet obtained the highest precision and specificity, with 97.23% and 90.2%, respectively. However, it achieved lower accuracy, 87.82%, and recall, 87.22%.

These metrics indicate that Mobilenet Unet had the smallest number of false negatives (21 exams or 2.60%) but a higher number of false positives (36 exams or 17.65%). Therefore, as missing a positive exam over a negative is more critical, Mobilenet Unet might be an efficient option to detect COVID-19 on MosMedData.

Then, to evaluate our architectures’ robustness, we performed external validation on the SPGC dataset, which was not on the training/validation/test sets, thus having a different distribution from our original images. Furthermore, the SPGC dataset has CAP exams, which were added to the “with lesion” class. Table 9 presents the results of all architectures evaluated in this work.

All architectures had similar and competitive results in the external validation on the SPGC dataset. Mobilenet Unet had the highest accuracy and F1-score, with 98.05% and 98.7%, respectively. Thus, we answer **RQ2**, as Mobilenet Unet detected exams with COVID-19 and CAP lesions and exams without lesions.

Mobilenet Unet is an intermediate architecture with a small encoder of only 3.4 million parameters and a decoder of 32 million parameters. Its size might have aided it in learning the task without overfitting samples with the same distribution from training/validation/test sets.

It is worth mentioning that external validation plays a vital role when comparing CNNs, because it simulates real-world situations, allowing us to choose the architecture that best generalizes for new samples.

### 4.4. COVID-19 and CAP Classification

We trained eight deep learning models on COVIDxCT to differentiate between COVID-19 and CAP CT slices. This classification distinguishes previously segmented lesions due to these two diseases, as our segmentation models cannot distinguish between COVID-19 and CAP lesions. We present our results in Table 10.

Our results of classifying CT slices as COVID-19 or CAP on COVIDxCT using eight different deep learning models are competitive. All the models achieved high accuracy, F1-score, precision, recall, and specificity. Among the models, Resnext101 achieved the highest overall performance, with accuracy of 96.79%, F1-score of 96.84%, precision of 94.71%, recall of 99.07%, and specificity of 94.52%. The performance of the other models is also noteworthy, with accuracy ranging from 94.84% to 96.79%. Finally, it is worth pointing out that the models’ specificity varied considerably, ranging from 91.22% to 95.32%.

Then, we externally validated these eight deep learning models on the slices with the most extensive lesions detected on the SPGC dataset, which can be lesions caused by COVID-19 or CAP. Finally, we summarize the results in Table 11.

These results indicate that the eight deep learning models we evaluated have promising potential for distinguishing COVID-19 from CAP using CT images. Overall, Densenet201 achieved the best performance with the highest accuracy, F1-score, and specificity. However, it is worth noting that the relatively low specificity for CAP means that the models may be more prone to false negatives in this class. This is an important consideration, as the accurate detection of Common-Acquired Pneumonia is also critical to the appropriate treatment and management of patients. It is important to note that these results were obtained by externally validating the models on a single slice from each CT scan from the SPGC dataset. Because the SPGC dataset has a smaller sample size than the COVIDxCT dataset used for model training, further evaluation on larger and more diverse datasets is needed to fully assess the generalizability and robustness of the models. Furthermore, to use these two-dimensional deep learning models and gain processing time, the three-dimensionality of the SPGC dataset CT scans was discarded, which also caused a loss of information.

**Figure 8:**
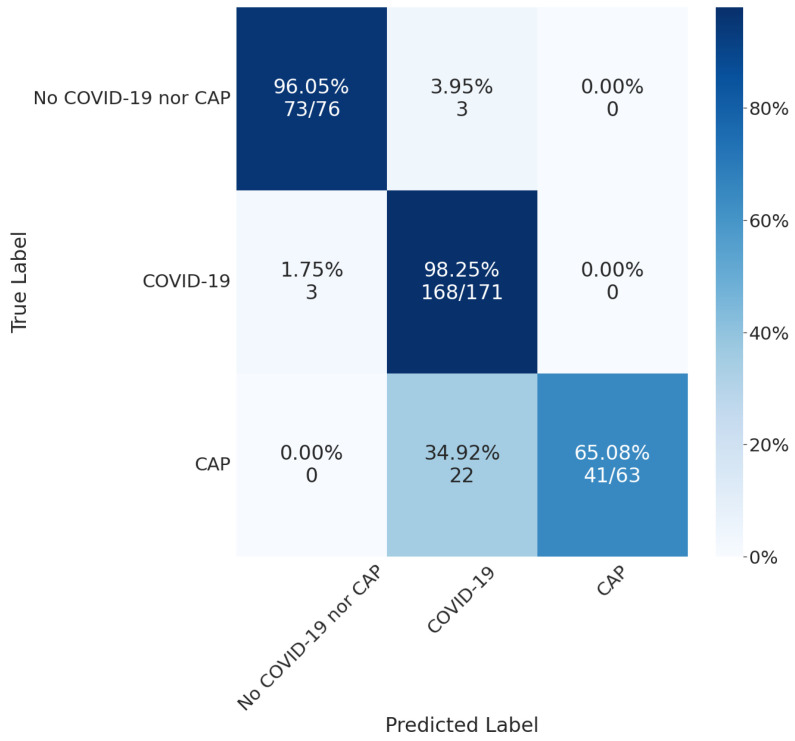
Final results using MobilenetV2 Unet for lesion detection and Densenet201 for COVID-19 or CAP classification.

By merging the segmentation, detection, and classification tasks, we obtained the confusion matrix in Figure 8. For lung segmentation, we applied Resnext101 Unet++; for lesion segmentation, we applied MobilenetV2 Unet; and for COVID-19 or CAP classification, we used Densenet201. These architectures were selected according to their overall results, mainly focusing on a low false-negative rate.

The confusion matrix shows that the classifier performed well in the COVID-19 class, with a high number of true positives (168) and a low number of false positives (3). However, there were some misclassifications, as 35% of CAP exams were classified as COVID-19. These results suggest that our classification models could not differentiate between the two classes or that there was insufficient information on the CT slice to differentiate between them.

**Figure 9:**
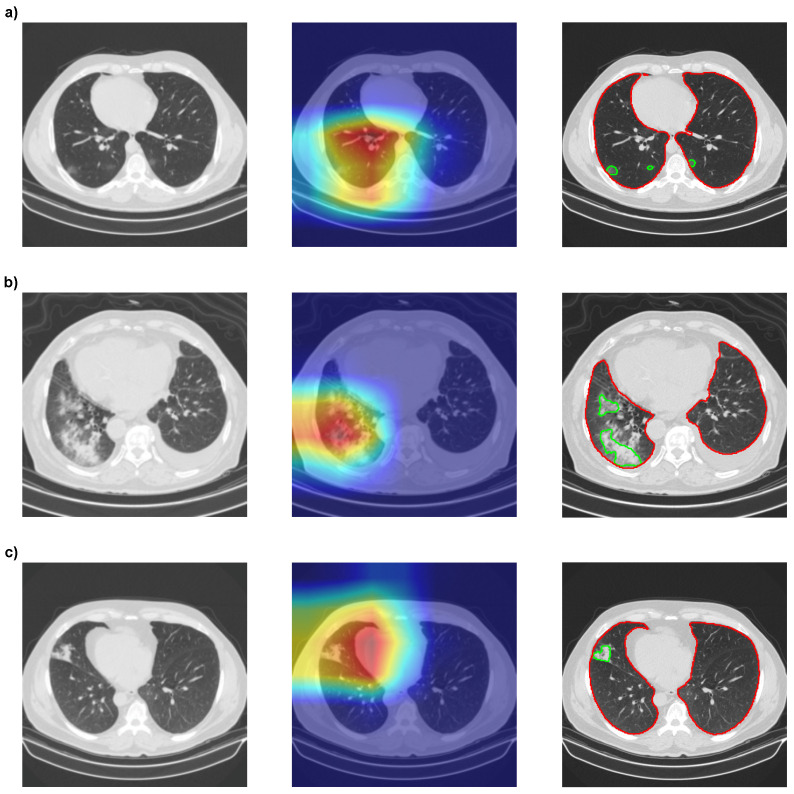
Comparison between the interpretability of the XAI method with Densenet201 and the segmentation method with MobilenetV2 Unet. (**a**) Correctly classified COVID-19-positive slice. (**b**) Correctly classified CAP-positive slice. (**c**) CAP-positive slice wrongfully classified as COVID-19. XAI is presented as heatmaps, with red representing the most important region of the image for classification and green the least important.

Then, we used GradCAMPlusPlus [56] to analyze the interpretability of our models with Densenet201. In Figure 9, we can see that the red spots, which highlight the most critical areas of the image for classification, mainly coincide with our segmentation results obtained with MobilenetV2 Unet.

The segmentation of COVID-19 or CAP lesions provides more information than the results of GradCAMPlusPlus, as it is possible to calculate the lesion area for each slice or full CT scan lesion volume. Furthermore, the GradCAMPlusPlus heatmap in Figure 9a might suggest that lesions only occurred in the left lung, while the segmentation showed lesions in both lungs. However, neither of the two methods could determine which specifications of the lesion were used by the CNNs to classify an image as COVID-19 or CAP. Thus, further interpretability is necessary, and **RQ3** is answered.

### 4.5. COVID-19 Severity

In order to provide numerical data about the segmented COVID-19 lesions, we calculated the severity of the disease based on the compromised area of the lungs. Then, we applied this methodology to MosMedData and compared the results. A summary is presented in Table 12.

**Figure 10:**
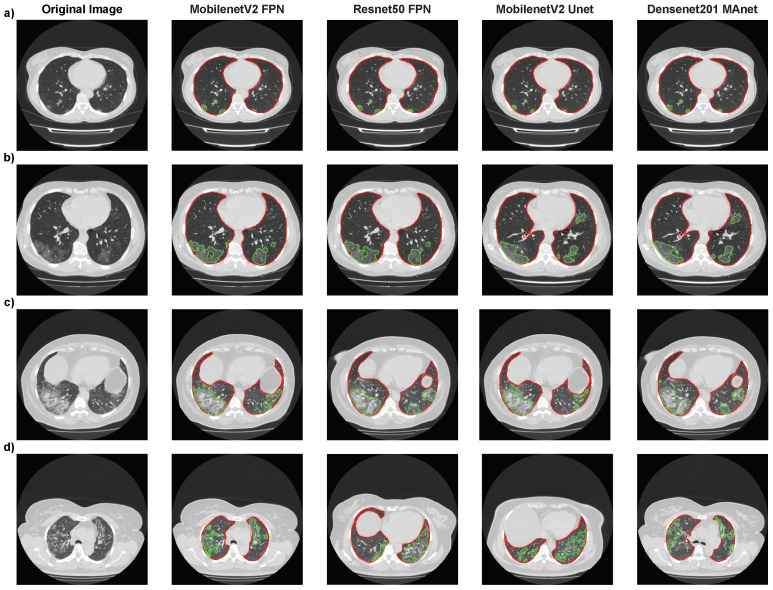
Segmentation results of Resnet50 FPN, Mobilenet FPN, Mobilenet Unet, and Densenet MAnet on MosMedData. Lung segmentation is represented by red contours, and lesion segmentation is represented by green contours. (**a**) Image from an exam of class 1. (**b**) Image from an exam of class 2. (**c**) Image from an exam of class 3. (**d**) Image from an exam of class 4.

Again, Mobilenet Unet obtained the highest results, with accuracy of 75.05%, F1-score of 73.26%, precision of 72.67%, and recall of 75.05%. Even if the metrics were not as high as for binary classification (“without lesion” or “with lesion”), Figure 10 shows, for four architectures (Resnet50 FPN, Mobilenet FPN, Mobilenet Unet, and Densenet MAnet), that our pipeline correctly segmented most lesions presented on the CT scans. The lower metrics obtained might have been due to the qualitative analysis made when labeling MosMedData, which we could not replicate with quantitative values.

We present the images where each model found the most extensive lesion area for that specific exam. The experiment found that all architectures could locate lesions in the same lung areas, indicating consistent performance. However, some architectures were unable to accurately identify certain lesion areas. Specifically, the MobilenetV2 FPN architecture failed to locate a small lesion in the right lung in the presented image (Figure 10a), while the other three architectures correctly identified it. These findings suggest that while all architectures performed similarly overall, there were still differences in their ability to accurately identify certain lesion areas, highlighting the importance of selecting the most suitable architecture for a specific task. These difficulties in detecting certain lesion areas could have worsened the results presented in the confusion matrices in Table 13.

Despite the success of our models in differentiating COVID-19 from non-COVID-19 cases (as shown in Table 8), we still observed a high degree of error when it came to distinguishing between different severity classes of COVID-19 on MosMedData. This error may have been due to several factors, such as the incorrect segmentation of lesions on CT scans by our models or the lack of quantified evaluation on MosMedData, as specialists qualitatively evaluated severity. This may have affected our results even when lesions were correctly segmented, thus answering **RQ4**.

According to our results, Resnet50 FPN was the fastest architecture on MosMedData, while Densenet201 MAnet was the slowest one. Specifically, the average time taken by Resnet50 FPN to segment lesions from all slices of MosMedData was 12.42 s. On the other hand, Resnext101 Unet++ took 17.43 s to segment lesions. Regarding the SPGC dataset, the fastest architecture was Mobilenet FPN, and the average time taken to segment lesions was 12.42 s. On the other hand, Densenet MAnet was the slowest, and the average time taken to segment lesions was 25.33 s. However, despite the speed difference, both models are viable for real-life usage. This means that the highlighted models can be effectively used in clinical settings, where speed and accuracy are essential and computational resources might be limited. The choice of model will depend on the user’s specific needs, such as the available computational resources.

### 4.6. Limitations

The first limitation of our work is that the high cost of CT scans and high exposure to ionizing radiation limits their widespread adoption in hospitals. This issue contributes to the reduced public data for training, and real-life testing and usage. Machine learning models identify patterns based on the data that they are trained on. Therefore, a machine learning model is biased when the data are biased. To partially address this issue, we conducted external validation on a new dataset obtained from the literature. However, since there are only a limited number of publicly available datasets, ensuring the model’s generalizability remains challenging.

Another limitation of this work is that all the architectures evaluated in this study were based on 2D images, whereas CT scans provide 3D information. Although using 2D images simplifies the computational complexity and reduces the training time required by the models, it may not fully capture the complexity of and variations in 3D structures. Consequently, the accuracy of the models in predicting and diagnosing various medical conditions using a 2D approach may be lower than that with a 3D approach. Another limitation of this study is that only analyzing the CT scan of a patient may not be sufficient for a diagnosis. CT scans provide useful information about the body’s internal structures, but they do not provide information about the patient’s symptoms or medical history. Therefore, integrating CT scan analysis with clinical data processed using natural language models could improve the accuracy of the diagnosis. By combining image and language models, physicians can make more informed decisions and provide better patient treatment options.

While our approach showed encouraging results, other CT scan factors that may contribute to the differentiation between CAP and COVID-19 might not be captured when separately analyzing only 2D slices. Moreover, it is known that there is a significant overlap in the imaging features of COVID-19 and other respiratory diseases, which makes differentiation challenging even with the use of advanced imaging techniques. Therefore, future studies using a more comprehensive approach that includes 3D imaging and clinical data may be necessary to improve the accuracy of COVID-19 diagnosis and further differentiate it from CAP.

Despite these limitations, the findings of this study provide valuable insights into the potential applications of deep learning and computer vision techniques in medical image analysis. Future studies can build upon these findings and further explore using 3D imaging and language models to improve medical diagnosis and treatment accuracy and efficiency.

## 5. Conclusions

In this work, we propose a deep learning-based approach to lung and lesion detection and segmentation, and COVID-19 and CAP classification using full CT scans. The results show that our pipeline correctly detected and segmented lesions due to COVID-19 and CAP in CT scans, differentiating these two classes from normal exams. In the classification task, we achieved competitive results in terms of accuracy, precision, recall, F1-score, and specificity on the COVIDxCT dataset. However, our metrics dropped when we performed external validation using the SPGC dataset, but they were still competitive. Our analysis obtained accuracy of 99.71 ± 0.05%, DSC score of 98.64 ± 0.19%, and HD of 3.9 ± 0.16% in lung segmentation with Resnext101 Unet++. In lesion segmentation, Densenet201 Unet++ achieved accuracy of 99.87 ± 0.01%, DSC score of 85.16 ± 1.13%, and HD of 3.4 ± 0.13%. When externally validating lesion detection on the SPGC dataset, using Resnetxt101 Unet++ for lung segmentation and Mobilenet Unet for lesion segmentation, we achieved accuracy of 98.05%, F1-score of 98.70%, precision of 98.7%, recall of 98.7%, and specificity of 96.05%, only needing 19.70 s per full CT scan. Finally, when classifying these detected lesions, Densenet201 reached accuracy of 90.47%, F1-score of 93.85%, precision of 88.42%, recall of 100.0%, and specificity of 65.07%.

Our pipeline can work as a CAD system to support healthcare professionals in monitoring disease progression over time, particularly in remote locations. It can be used in junction with portable CT scanners to reach populations in remote areas and integrate our pipeline with telemedicine for remote monitoring.

Nevertheless, this work did not exhaust the possibilities of researching COVID-19 and CAP detection, segmentation, and classification. In future works, one might evaluate the trade-off between processing time and accuracy using 3D segmentation and classification architectures. In addition, clinical data can be used to aid in differentiating COVID-19 and CAP CT exams. Another possible approach to improving disease detection is to increase monitored data. For instance, Natural Language Processing models can extract clinical data from patient records. It could yield valuable information to be incorporated into the CAD system as significant features. This combination of patient information could enhance the classification of the two diseases.

## Figures and Tables

**Table 1:** Related works.

Work	Seg.	Classification	Datasets	External Validation?	Metrics
Harmon et al. [16]	AH-Net	Densenet121-based	Private	**X**	Accuracy, sensitivity, and specificity
Varan et al. [17]	Threshold and region growing	ResNet50, ResNet101, DenseNet169, and DenseNet201	MosMedData	**X**	Accuracy
Li et al. [18]	U-net	Resnet50-based	Private	**X**	Sensibility, specificity, and AUC
Hasan et al. [19]	Threshold and morphological operations	Proposed classification: 3D CNN-based	MosMedData	**X**	AUC
Abdel-Basset et al. [20]	U-net-based	EfficientNet-B7-based	COVID-CT-MD	**X**	Accuracy, DSC (F1), Jaccard index, and AUC
Zhang et al. [23]	U-net, DRUNET, FCN, and SegNet	3D ResNet-18-based			Accuracy and, AUROC
Amyar et al. [24]	Encoder–decoder-based	Alexnet, VGG-16, VGG-19, ResNet50, DenseNet169, InceptionV3, Inception-ResNet v2, and Efficient-Net	COVID-CT-MD, HBCC, and MedSeg	**X**	Accuracy, DSC, sensibility, specificity, and AUC
Qiblawey et al. [25]	ED-CNNs, UNet, and FPN	Lesion segmentation and threshold	COVID-CT, CTDATA (Kaggle), and MosMedData	**✓**	DSC, IoU, sensitivity, and specificity
Wang et al. [26]	Encoder–decoder-based	**X**	Private	**X**	DSC, RVE, and HD95
Zhou et al. [27]	Proposed	**X**	Harbin and private	**X**	DSC and recall
This work	Resnext101 Unet++ and MobilenetV2 Unet	Densenet201	Coronacases, Kaggle, Medical Segmentation, MosMedData, COVIDxCT, and SPGC	**✓**	Accuracy, F1 (DSC), HD, precision, recall, and specificity

**Table 2:** Datasets used for each task.

Database	Task	No. of COVID-19 Exams	No. of CAP Exams	No. of Non-COVID-19, Non-CAP Exams	No. of Total Images
Coronacases	Lung segmentation	10	0	0	2581
Kaggle	Lung/lesion segmentation	0	0	n/a	267
Medical Seg.	Lung segmentation	9	0	0	829
Coronacases	Lesion segmentation	10	0	0	2156
Medical Seg	Lesion segmentation	9	0	0	713
Mosmed Seg	Lesion segmentation	50	0	50	3357
Mosmed Seg	Validation	806	0	50	42,224
COVIDxCT	Image classification	3731	932	0	353,536
SPGC	External validation	171	60	71	46,024

**Table 3:** Data augmentation techniques and parameters.

Method	Task	Parameters
HorizontalFlip	Lung/lesion segmentation	${p\;}^{1}$ = 0.5
ShiftScaleRotate	Lung/lesion segmentation	Shift limit = 0.05, scale limit = 0.1, rotate limit = 15, and *p* = 0.5
RGBShift	Lung segmentation	r shift limit = 25, g shift limit = 25, b shift limit = 25, and *p* = 0.5
RandomBrightnessContrast	Lung/lesion segmentation	Brightness limit = 0.3, contrast limit = 0.3, and *p* = 0.5

^1^ *p* is the probability of applying the method to the image.

**Table 4:** Grid search parameters.

Hyperparameters	Task	Parameters
Batch size	Lesion segmentation	(8, 16, 32, 64)
Epochs	Lesion segmentation	(25, 50, 75)
Learning rate	Lesion segmentation	(0.001, 0.0001, 0.00001)
Encoder	Lesion segmentation	(mobilenet, resnet50, densenet201, resnext101)
Decoder	Lesion segmentation	(FPN, Unet, Unet++, MAnet)
Patience	Lesion segmentation	(5, 10, 15)
Loss	Lesion segmentation	(Lovasz, Dice, Tversky)
Tversky beta	Lesion segmentation	(0.3, 0.4, 0.6, 0.7, 0.8, 0.9)
Optimizer	Lesion segmentation	(Adam, RMSprop)

**Table 5:** Lesion segmentation-optimized hyperparameters.

Architecture	Batch Size	Epochs	Loss	Beta	LR	Optimizer	Patience
MobilenetV2 FPN	16	75	Dice	n/a	0.0001	Adam	15
Resnet50 FPN	64	50	Dice	n/a	0.0001	RMSprop	15
Densenet201 FPN	16	75	Dice	n/a	0.0001	RMSprop	15
Resnext101 FPN	64	75	Tversky	0.9	0.001	Adam	10
MobilenetV2 Unet	64	50	Tversky	0.3	0.001	Adam	15
Resnet50 Unet	64	50	Tversky	0.7	0.0001	RMSprop	10
Densenet201 Unet	32	75	Tversky	0.3	0.00001	Adam	15
Resnext101 Unet	64	75	Lovasz	n/a	0.0001	Adam	10
MobilenetV2 Unet++	32	50	Dice	n/a	0.0001	Adam	15
Resnet50 Unet++	32	75	Lovasz	n/a	0.00001	RMSprop	10
Densenet201 Unet++	32	75	Tversky	0.3	0.00001	Adam	15
Resnext101 Unet++	16	50	Lovasz	n/a	0.0001	Adam	15
MobilenetV2 MAnet	32	25	Tversky	0.7	0.0001	RMSprop	5
Resnet50 MAnet	64	50	Lovasz	n/a	0.00001	RMSprop	5
Densenet201 MAnet	32	75	Dice	n/a	0.00001	RMSprop	15
Resnext101 MAnet	16	75	Lovasz	n/a	0.0001	Adam	15

**Table 6:** Lung segmentation results.

Architecture	Acc (%)	F1 (DSC) (%)	HD
MobilenetV2 FPN	99.55 ± 0.05	97.9 ± 0.16	4.4 ± 0.1
Resnet50 FPN	99.62 ± 0.04	98.25 ± 0.15	4.19 ± 0.15
Densenet201 FPN	99.61 ± 0.06	98.21 ± 0.2	4.2 ± 0.14
Resnext101 FPN	99.63 ± 0.06	98.29 ± 0.2	4.18 ± 0.12
MobilenetV2 Unet	99.63 ± 0.08	98.29 ± 0.31	4.1 ± 0.21
Resnet50 Unet	99.7 ± 0.05	98.59 ± 0.17	3.92 ± 0.15
Densenet201 Unet	99.69 ± 0.06	98.56 ± 0.21	3.96 ± 0.17
Resnext101 Unet	99.7 ± 0.05	98.61 ± 0.17	3.92 ± 0.15
MobilenetV2 Unet++	99.67 ± 0.05	98.46 ± 0.2	4.0 ± 0.12
Resnet50 Unet++	99.69 ± 0.05	98.58 ± 0.18	3.95 ± 0.14
Densenet201 Unet++	99.7 ± 0.05	98.63 ± 0.19	3.93 ± 0.18
Resnext101 Unet++	99.71 ± 0.05	98.64 ± 0.19	3.9 ± 0.16
MobilenetV2 MAnet	99.66 ± 0.05	98.41 ± 0.17	4.01 ± 0.12
Resnet50 MAnet	99.68 ± 0.05	98.51 ± 0.18	3.99 ± 0.14
Densenet201 MAnet	99.66 ± 0.05	98.42 ± 0.17	4.03 ± 0.13
Resnext101 MAnet	99.69 ± 0.05	98.54 ± 0.17	3.96 ± 0.14

**Table 7:** Lesion segmentation results.

Architecture	Acc (%)	F1 (DSC) (%)	HD
MobilenetV2 FPN	99.85 ± 0.01	82.95 ± 1.45	**2.86 ± 0.12**
Resnet50 FPN	99.86 ± 0.01	83.84 ± 1.13	4.0 ± 0.2
Densenet201 FPN	99.86 ± 0.01	83.47 ± 1.01	2.87 ± 0.1
Resnext101 FPN	99.84 ± 0.02	82.17 ± 1.71	4.28 ± 0.25
MobilenetV2 Unet	99.86 ± 0.01	82.59 ± 1.32	4.1 ± 0.21
Resnet50 Unet	99.87 ± 0.02	84.55 ± 1.32	4.06 ± 0.26
Densenet201 Unet	**99.87 ± 0.01**	84.8 ± 1.1	3.88 ± 0.22
Resnext101 Unet	99.85 ± 0.02	82.75 ± 2.1	4.21 ± 0.2
MobilenetV2 Unet++	99.87 ± 0.02	84.51 ± 1.08	3.95 ± 0.26
Resnet50 Unet++	**99.87 ± 0.01**	84.41 ± 1.32	3.48 ± 0.12
Densenet201 Unet++	**99.87 ± 0.01**	**85.16 ± 1.13**	3.4 ± 0.13
Resnext101 Unet++	99.86 ± 0.01	83.72 ± 1.26	2.87 ± 0.14
MobilenetV2 MAnet	99.83 ± 0.02	80.9 ± 1.34	3.77 ± 0.13
Resnet50 MAnet	99.85 ± 0.01	82.37 ± 1.14	4.11 ± 0.27
Densenet201 MAnet	99.86 ± 0.01	83.81 ± 1.01	3.52 ± 0.19
Resnext101 MAnet	99.86 ± 0.01	83.18 ± 1.16	2.86 ± 0.13

**Table 8:** COVID-19 lesion detection external validation on MosMedData.

Architecture	Acc (%)	F1 (%)	Prec (%)	Rec (%)	Spec (%)	Time per Exam (s)
MobilenetV2 FPN	91.88	94.94	94.36	95.53	77.45	12.69
Resnet50 FPN	90.40	94.0	93.71	94.29	75.0	**12.42**
Densenet201 FPN	90.89	94.43	92.20	96.77	67.65	15.05
Resnext101 FPN	91.39	94.68	93.37	96.03	73.04	14.68
MobilenetV2 Unet	**94.36**	**96.5**	95.62	**97.39**	82.35	12.88
Resnet50 Unet	92.48	95.31	94.84	95.78	79.41	12.79
Densenet201 Unet	90.40	94.08	92.56	95.66	69.61	13.61
Resnext101 Unet	91.09	94.55	92.32	96.9	68.14	12.54
MobilenetV2 Unet++	92.18	95.05	95.95	94.17	84.31	12.44
Resnet50 Unet++	90.40	93.87	95.62	92.18	83.33	13.03
Densenet201 Unet++	91.78	94.94	93.40	96.53	73.04	12.98
Resnext101 Unet++	91.88	94.99	93.61	96.40	74.02	14.94
MobilenetV2 MAnet	90.99	94.38	93.97	94.79	75.98	14.05
Resnet50 MAnet	87.92	92.49	91.81	93.18	67.16	14.34
Densenet201 MAnet	87.82	91.96	**97.23**	87.22	**90.2**	17.43
Resnext101 MAnet	91.88	94.94	94.47	95.41	77.94	16.87

**Table 9:** Lesion detection external validation on the SPGC dataset.

Architecture	Acc (%)	F1 (%)	Prec (%)	Rec (%)	Spec (%)	Time per Exam (s)
MobilenetV2 FPN	97.39	98.28	97.85	98.70	93.42	**19.03**
Resnet50 FPN	95.11	96.82	95.0	98.7	84.21	19.49
Densenet201 FPN	96.42	97.64	96.61	98.70	89.47	21.71
Resnext101 FPN	97.39	98.28	97.85	98.70	93.42	21.45
MobilenetV2 Unet	**98.05**	**98.70**	98.7	98.7	96.05	19.70
Resnet50 Unet	97.39	98.28	97.45	**99.13**	92.11	21.45
Densenet201 Unet	94.79	96.61	94.61	98.70	82.89	21.71
Resnext101 Unet	91.53	94.63	90.51	**99.13**	68.42	22.72
MobilenetV2 Unet++	97.72	98.47	99.12	97.84	**97.37**	19.22
Resnet50 Unet++	95.77	97.12	**99.55**	94.81	98.68	23.01
Densenet201 Unet++	97.39	98.28	97.45	**99.13**	92.11	23.29
Resnext101 Unet++	94.79	96.61	94.61	98.70	82.89	23.29
MobilenetV2 MAnet	95.11	96.82	95.0	98.70	84.21	20.26
Resnet50 MAnet	96.42	97.58	99.11	96.10	**97.37**	22.93
Densenet201 MAnet	94.14	96.22	93.47	**99.13**	78.95	25.33
Resnext101 MAnet	95.44	97.03	95.02	**99.13**	84.21	21.45

**Table 10:** COVID-19 and CAP classification results on COVIDxCT.

Architecture	Acc (%)	F1 (%)	Prec (%)	Rec (%)	Spec (%)
Mobilenet	95.85	95.91	94.12	97.78	93.94
Resnet50	95.79	95.90	93.09	98.88	92.73
Densenet201	96.43	96.48	94.66	98.37	94.50
Resnext101	**96.79**	**96.84**	94.71	99.07	94.52
Squeezenet	94.84	95.00	91.75	98.49	91.22
Efficientnet	96.18	96.20	**95.36**	97.04	**95.32**
Shufflenet	95.69	95.78	93.35	98.35	93.05
Ghostnet	96.18	96.28	93.42	**99.32**	93.06

**Table 11:** COVID-19 and CAP classification external validation on the SPGC dataset.

Architecture	Acc (%)	F1 (%)	Prec (%)	Rec (%)	Spec (%)
Mobilenet	87.44	91.87	86.77	97.61	60.31
Resnet50	86.58	91.50	84.77	99.40	52.38
Densenet201	**90.47**	**93.85**	**88.42**	**100.0**	**65.07**
Resnext101	88.31	92.43	87.30	98.21	61.90
Squeezenet	86.14	91.20	84.69	98.80	52.38
Efficientnet	89.17	93.03	87.43	99.40	61.90
Shufflenet	87.87	92.26	86.08	99.40	57.14
Ghostnet	88.74	92.73	87.36	98.80	61.90

**Table 12:** COVID-19 severity on MosMedData.

Architecture	Acc (%)	F1 (%)	Prec (%)	Rec (%)
MobilenetV2 FPN	69.9	69.95	70.27	69.9
Resnet50 FPN	71.98	70.77	70.52	71.98
Densenet201 FPN	67.13	67.81	69.07	67.13
Resnext101 FPN	69.5	69.68	70.27	69.5
MobilenetV2 Unet	**75.05**	**73.26**	**72.67**	**75.05**
Resnet50 Unet	72.67	71.08	70.25	72.67
Densenet201 Unet	66.93	67.49	68.42	66.93
Resnext101 Unet	69.41	69.07	69.58	69.41
MobilenetV2 Unet++	72.97	71.09	70.3	72.97
Resnet50 Unet++	69.21	68.7	68.79	69.21
Densenet201 Unet++	72.18	70.86	70.83	72.18
Resnext101 Unet++	70.89	70.4	70.44	70.89
MobilenetV2 MAnet	70.89	70.25	70.29	70.89
Resnet50 MAnet	66.93	66.26	65.99	66.93
Densenet201 MAnet	66.44	66.12	67.28	66.44
Resnext101 MAnet	71.09	70.58	70.47	71.09

**Table 13:** Confusion matrix results of Resnet50 FPN, Mobilenet FPN, Mobilenet Unet, and Densenet MAnet on MosMedData.

True Class	Classified as	MobilenetV2 FPN	Resnet50 FPN	MobilenetV2 Unet	Densenet201 MAnet
CT-0	CT-0	158 (77%)	153 (70%)	168 (82%)	184 (90%)
	CT-1	46 (23%)	50 (25%)	36 (18%)	20 (10%)
	CT-2	0	1	0	0
	CT-3	0	0	0	0
	CT-4	0	0	0	0
CT-1	CT-0	35 (6%)	45 (7%)	21 (3%)	100 (16%)
	CT-1	506 (80%)	534 (84%)	559 (88%)	452 (71%)
	CT-2	59 (9%)	37 (6%)	36 (6%)	52 (52%)
	CT-3	14 (2%)	5 (1%)	8 (1%)	12 (2%)
	CT-4	20 (3%)	13 (2%)	10 (2%)	18 (3%)
CT-2	CT-0	1 (1%)	1 (1%)	0	2 (1%)
	CT-1	80 (63%)	82 (65%)	94 (74%)	83 (66%)
	CT-2	34 (28%)	35 (28%)	26 (21%)	25 (20%)
	CT-3	8 (6%)	5 (4%)	4 (3%)	10 (8%)
	CT-4	3 (2%)	3 (2%)	2 (2%)	6 (5%)
CT-3	CT-0	0	0	0	1 (2%)
	CT-1	21 (48%)	18 (41%)	22 (50%)	18 (41%)
	CT-2	9 (20%)	15 (34%)	13 (30%)	10 (23%)
	CT-3	7 (16%)	3 (7%)	4 (9%)	9 (20%)
	CT-4	7 (16%)	8 (18%)	5 (11%)	6 (14%)
CT-4	CT-0	0	0	0	0
	CT-1	0	0	0	0
	CT-2	0	0	1 (50%)	1 (50%)
	CT-3	1 (50%)	0	0	0
	CT-4	1 (50%)	2 (100%)	1 (50%)	1 (50%)

## Data Availability

All used datasets are publicly available and can be obtained online.
